# Clinical Cases

**Published:** 1907-08-03

**Authors:** 


### Practical Demonstrations.

### CLINICAL CASES

### Osteo-arthritis in a Young Boy.

Although there is no family arthritic history, this boy, aged three years and two months, presents a multiple joint affection to which we are justified in giving the name of osteo-arthritis or rheumatoid arthritis, neither of them very satisfactory terms. He developed normally, though bottle-fed, cutting his first teeth at seven months and walking at sixteen months. In the second year of life he had a long illness from whooping cough and pneumonia. Six months ago he had an ulcerated mouth, and now you notice that he has very bad teeth. For seven weeks he has got pale, and been easily tired. The joint swelling, of wrists and ankles, is of twenty-one months' duration, dating from the whooping-cough. For four to five months he has had pain and stiffness in the neck. Lately the knees have become a little involved. On examination it is found that the joint swelling is due to peri-arthritis, that the bones are not affected, and that there are no osteophytic deposits. Small palpable glands are present in the axillæ and groins. The spleen is not enlarged. The slight glandular enlargement is partly due to toxæmia, partly apparent because the child is thin. Little importance can be attached to enlargement of glands or spleen in children, for it occurs on such slight provocation.

### Monoplegia due to Chorea.

There is considerable loss of power in the right arm of this five-year-old girl, a not uncommon condition in chorea. Such palsy may precede, coincide with, or follow an attack. In the present case choreic movements were noticed before the paresis. They followed three days after the child had been "frightened by a dog." A history of fright is not uncommon, and terror sometimes appears to be the true exciting factor. The child is one of six children. No one in the family, except the mother, has had rheumatic fever or other rheumatic signs. The father is epileptic. Thus there is both a rheumatic and a neurotic heredity, and it is impossible to be quite certain to which of these the child owes her chorea. The child has always been healthy, free from rheumatism, and has no endocarditis. I am by no means convinced that all cases are rheumatic. The paralysis may be so severe and extensive as to render the choreic movements unnoticeable. Such a case may be diagnosed as one of acute anterior poliomyelitis, especially if the knee jerks are absent. The knee jerks in chorea may be exaggerated, decreased, or absent. Occasionally they disappear quite suddenly during the course of the disease, even though neither arsenic nor bromide is being given. Another peculiarity is the "hung-up" type of knee jerk. Unfortunately the child is too emotional to permit me to demonstrate it satisfactorily. The feature of it is that the limb gradually rises to a position of extreme extension and rigidity, and remains so for a few seconds. This peculiarity is due to prolonged or choreic contraction of the rectus femoris. This child's speech is unaffected.

### Cerebral Diplegia and Imbecility.

The last patient is a boy, in his fourth year, well grown for his age, the third of four children. The family history is of negative value. The child was small and very feeble at birth; labour was easy and rapid. On the whole it is probable that the child was premature. He did not cry for fourteen days, and lay with arms and legs crossed. At four months he began to kick his legs about. The first teeth were cut at seven months, but the child could not hold his head up until eighteen months, and did not sit up until a year later. He has never fed himself, is not clean in his habits, cannot talk, but is said to understand. Certainly he is not quite an idiot. Sight and hearing are good, and he enjoys food. The facial aspect is somewhat imbecile. The head is rachitic in type. The arms are rather weak and athetoid in movement. The legs are weak, approximated unduly, and a trifle rigid. The reflexes are much exaggerated. The gait, when he is supported, is somewhat spastic, knees and ankles knocking together, but does not show the "cross-legged" type of progression.

There is no doubt that this is a case of severe cerebral mischief, and that it belongs to the group of cerebral palsies or birth palsies, of the spastic type, sometimes called Little's disease. It may depend on immaturity of the nerve cells in the cortex or the motor tracts; on degenerative changes, secondary to injury at birth or meningeal hæmorrhage; meningo-encephalitis, due to septic or other infection before, during, or after birth; or to gross cerebral defect. It is quite impossible to state the exact cause in this particular instance. At any rate it is not one of the cases of simple immaturity, for there is too great impairment of intelligence, lack of cleanliness, and absence of improvement. The prognosis is hopeless. Roughly, the prospect of improvement in these cases varies directly as the degree of intelligence. If the latter is good, recovery may in time be almost, if not quite, complete.

---

The Swiss Medical Congress.—Neuchâtel, which, although not a University town, endows an Academy of Natural Sciences, welcomed the delegates of the Swiss Medical Congress this year. The meetings of the Congress have been of more than usual interest. Professor Baraneck gave a demonstration of his tuberculin, and some of his co-workers exhibited cases treated by his tuberculin injections. In all these eminently encouraging results had been obtained. Dr. Borel detailed the history of an interesting case of psychical blindness of 20 years' duration. The disease had had a sudden onset, and had been attended with transient hemiplegia. Charcot, who saw the patient shortly before his death, had been greatly interested in the case, which had presented some unusually interesting features, but the great scientist died before he had been able definitely to arrive at a diagnosis. Other papers of general interest were read, one of the most interesting being the opening address of Dr. de Reignier, the doyen of the local practitioners, who reviewed the advance of surgery and medicine in Switzerland during the last decade, and pointed out that while much had been done to alleviate physical suffering, comparatively little had been done in the way of lessening moral suffering. A feature of the Congress was the spontaneous tribute which the delegates paid to the work of Lord Lister.

